# Supplementary material for: Conditional chemoconnectomics (cCCTomics) as a strategy for efficient and conditional targeting of chemical transmission
Source: eLife. 2024 Apr 30;12:RP91927. doi: 10.7554/eLife.91927 (PMC11060718; doi:10.7554/eLife.91927)
Supplement: Figure 3—source data 3. [file elife-91927-fig3-data3.zip › Figure 3 source data3 part 1.zip!/Figure 3-Source data 3-part1/1071f2-1-CRISPResso_Report_ztwktvfp.zip!/CRISPResso_on_1071f2_1/1c.Alignment_barplot.pdf]

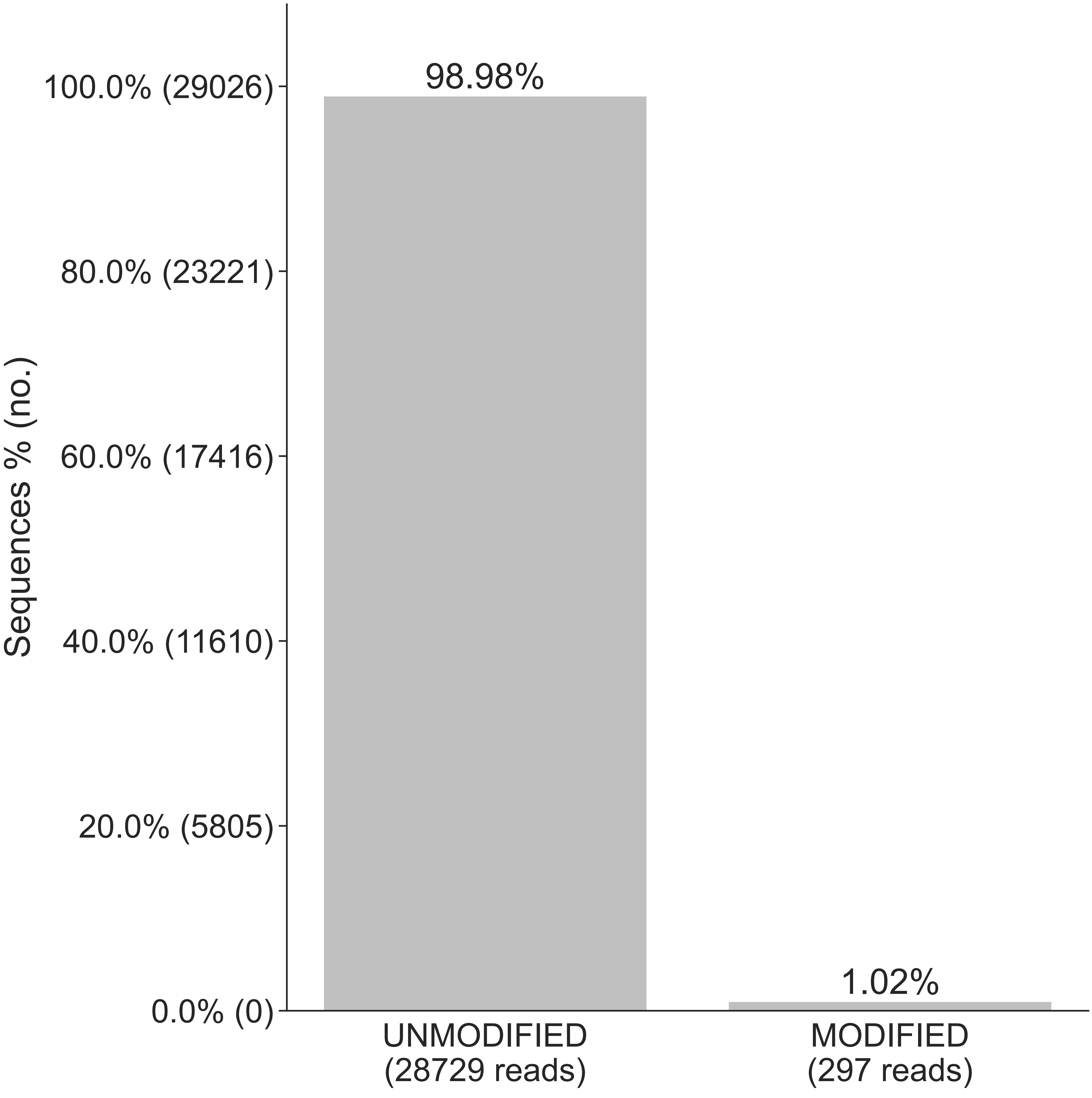
Sequences % (no.)
100.0% (29026)
80.0% (23221)
60.0% (17416)
40.0% (11610)
20.0% (5805)
0.0% (0)
98.98%
1.02%
UNMODIFIED
(28729 reads)
MODIFIED
(297 reads)
